# Enhanced LC-ESI-MS/MS Sensitivity by Cationic Derivatization of Organophosphorus Acids

**DOI:** 10.3390/molecules28166090

**Published:** 2023-08-16

**Authors:** Tamar Shamai Yamin, Moran Madmon, Ariel Hindi, Avital Shifrovich, Hagit Prihed, Merav Blanca, Avi Weissberg

**Affiliations:** Department of Analytical Chemistry, Israel Institute for Biological Research (IIBR), P.O. Box 19, Ness Ziona 7410001, Israel

**Keywords:** organophosphorus acids, cationic derivatization, CAX-B, LC-MS/MS, diagnostic ions

## Abstract

The chemical derivatization to enhance the signal intensity and signal-to-noise (S/N) of several organophosphorus (OP) acids in liquid chromatography tandem mass spectrometry (LC-ESI-MS/MS) is illustrated. The OP class of compounds represents the environmental degradants of OP nerve agents and pesticides. N-(2-(bromomethyl)benzyl)-N,N-diethylethanaminium bromide (CAX-B) was utilized to derivatize a panel of eight acids consisting of five alkyl methylphosphonic acids (ethyl-, isopropyl-, isobutyl-, cyclohexyl-, and pinacolyl-methylphosphonic acid) along with three dialkylphosphate analogs (diethyl-, dibutyl-, and diethyl thio-phosphate). The derivatization reaction with CAX-B was conducted in acetonitrile in the presence of potassium carbonate at 70 °C for 1 h. The resulting acid derivatives were analyzed with an LC-Orbitrap-ESI-MS/MS, and their dissociation processes were investigated. It was found that the derivatization procedure increased the limits of identification (LOIs) by one to over two orders of magnitude from the range of 1 to 10 ng/mL for the intact OP-acids to the range of 0.02–0.2 ng/mL for the derivatized acids utilizing an LC-MS(QqQ) in MRM mode, regardless of the sample matrix (hair, concrete, or plant extracts). The interpretation of the corresponding ESI-MS/MS spectra for each type of derivatized sub-OP family revealed the formation of characteristic neutral losses and a characteristic ion for the organophosphorus core. This derivatization is beneficial and useful for screening and identifying target and “unknown” OP acids.

## 1. Introduction

Organophosphorus (OP) nerve agents, such as V- and G-nerve agents, are extremely toxic compounds, and the threat they pose is well understood and recognized. These agents have relatively short lifespans in the human body and in the environment because they are hydrolyzed or metabolized to stable alkyl methylphosphonic acids (AMPAs) [1]. Because these acids originate almost exclusively from nerve agent hydrolysis, they are used as indicators for specific nerve agent exposure and use. In contrast to nerve agents, OP pesticides are considered less toxic compounds, are widely used for agriculture, and are considered one of the most common environmental pollutants. Despite the apparent benefits of OP pesticide use, these compounds cause severe toxicity and death from acute poisoning worldwide [2]. In addition, the accumulation of these highly toxic agents in water supplies and food products increases the risks of chronic human exposure. OP pesticides are classified as non-persistent pesticides with a relatively short lifetime, e.g., the half-life of dicrotophos and profenfos is a few days in soil [3,4]. Their urinary metabolites and environmental degradation products are widely used for assessing OP pesticide exposure [5]. Currently, dozens of OP pesticides are registered for use in agriculture, most of which are O,O-dialkyl substituted. Their degradation products and metabolites are dialkyl phosphate (DAP), dialkyl thiophosphate (DATP), and dialkyl-dithiophosphate (DADTP). Hence, it is difficult to identify individual pesticides from these degradation products or metabolites, as they can provide information only on the OP pesticide class. Direct detection of these OP hydrolysis products with a GC-MS is not suitable because these compounds are not sufficiently volatile. Therefore, several methods for their derivatization have been developed, including silylation [6] and alkylation [7,8,9]. LC-ESI-MS/MS is often the most appropriate and powerful technique for the determination of polar, nonvolatile OP compounds and OP-degradation products, allowing their direct analysis in aqueous samples with little or no sample preparation [10,11]. Negative electrospray ionization (ESI) is the first choice for their detection and identification; however, positive ESI is also feasible for some of them. However, the limits of detection (LODs) of OP acids are relatively high [10,12], and moreover, the presence of interfering background chemicals mostly in the lower mass range may induce ion suppression or inferior peak shape, rendering the identification of such OP acids difficult in reversed-phase columns.

Designated laboratories involved in off-site analysis aim to enhance their analytical capabilities to meet the challenges associated with the verification of OP nerve agent indicators (e.g., AMPAs). This challenge becomes more difficult in the presence of high matrix interferences with low concentration levels of analytes [13]. In addition, there is a growing interest among public health organizations in the assessment of community exposures to OP pesticides in environmental matrices and in food [14,15].

To overcome these limitations, various methods have been developed to enhance the sensitivity of these OP acids. Some of them include the use of dicationic or tricationic reagents [16,17,18] post-LC to form ion pairs with OP acids, chemical derivatization using RP [19,20] or hydrophilic interaction liquid chromatography (HILIC) chemistry [21], and most recently, mixed mode chromatography in combination with postcolumn dicationic ion-pairing-based LC-MS for dialkyl phosphate analysis [22]. However, ion-pairing additives have been proven to be detrimental for LC-MS determination [12]. In our previous study [20], 3-(bromoacetyl)pyridine was used as a derivatizing agent for the selective determination of various OP acids in LC-ESI-MS/MS, enhancing the signal intensity by 10–50-fold only when relatively high concentrations of OP acids were utilized (above 10 ng/mL). Moreover, the derivatization reaction efficiency was highly affected by matrix interferences, and no improvement in signal-to-noise intensity was observed.

This study is aimed to develop a qualitative technique to enhance the identification sensitivity of targeted environmentally relevant OP acids related to chemical warfare agents and pesticides utilizing chemical derivatization with N-(2-(bromomethyl)benzyl)-N,N-diethylethanaminium bromide (CAX-B) followed by LC-ESI-MS/MS. In addition, the interpretation of the mass spectral data and the determination of characteristic ions may assist both in sensitivity enhancement and the unambiguous identification of “unknown” OP acids.

## 2. Results and Discussion

### 2.1. Derivatizing Agent Design

This study focused on the development of a generic and sensitive identification method for OP acids related to nerve agents and pesticides (ethyl methylphosphonic acid (EMPA), isopropyl methylphosphonic acid (IMPA), isobutyl methylphosphonic acid (IBMPA), cyclohexyl methylphosphonic acid (CMPA), pinacolyl methylphosphonic acid (PMPA), diethyl phosphate (DEP), dibutyl phosphate (DBP), and diethyl thiophosphate (DETP)) using an LC-ESI-MS/MS. In order to enhance sensitivity, the derivatization product of the OP-acids should possess a high proton-affinity moiety (cationic tag), which is highly responsive in positive ESI-MS mode. In addition, the derivatization product should fragment efficiently upon collision-induced dissociation (CID) and produce intense and diagnostic product ions for sensitive MS/MS detection and unambiguous identification. These diagnostic ions may be used for structural core-based screening methods. We chose to use the CAX-B derivatizing agent to generate precursor ions (possessing a cationic tag) and characteristic product ions (possessing a tropylium/benzyl cation), which are highly responsive in positive ESI-MS/MS mode. CAX-B was first studied by Wang et al. [23] and found to be an efficient derivatizing agent for analytes containing aromatic hydroxyl groups and more recently was applied for cyanide determination with an LC-MS [24].

### 2.2. Optimization of the Reaction between the OP Acids and CAX-B

**Figure 1:**
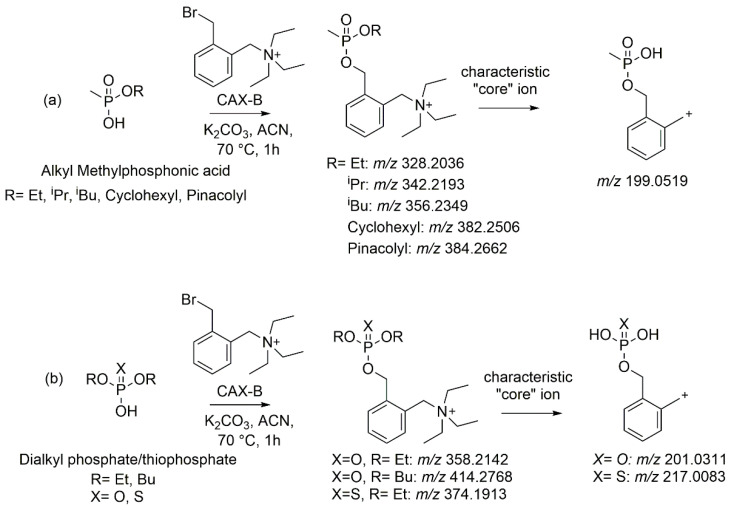
Reaction scheme of the OP acids (AMPAs (**a**) and dialkyl phosphate/thiophosphate (**b**)) after derivatization with CAX-B, along with the diagnostic product ion of the core structure of each OP family derived from the ESI-MS/MS spectra.

The conventional derivatization reaction of an OP-acid with a benzyl halide (e.g., pentafluorobenzyl bromide) for GC analysis [25] or LC-MS analysis, as we previously reported [20], requires the use of a base, typically sodium or potassium carbonate, to deprotonate the OP-acid followed by the reaction at 70 °C. We applied the aforementioned procedure, utilizing an acidic chromatographic method that we recently developed for the cyanide derivative (CAX-CN) [24], which resulted in proper peak shapes (peak height and width) and optimized the reaction between CAX-B and OP acids. Initial experiments revealed low yields when water was used as a solvent, probably due to the hydrolysis of the CAX-B reagent by a hydroxyl group, which is a better nucleophile than the phosphonate anion. Therefore, we selected the nonprotic polar solvent acetonitrile, and potassium carbonate was selected as the base (1 mg/mL). The influences of reaction time (1, 2, and 3 h), temperature (25, 50, and 70 °C), and CAX-B concentration (0.01, 0.1, and 1 mg/mL) on the conversions of the OP acids to the corresponding OP derivatives were investigated and optimized, one variable at a time. No increase in signal intensities of the CAX-OP acids was observed beyond 1 h at 70 °C. After a 1 h reaction at 25 °C, conversion yields were negligible. At 70 °C, more than 95% was converted, which was slightly higher than observed at 50 °C. A large amount of derivatizing agent (1 mg/mL) was added to ensure that an excess of reagent was present. Decreasing its concentration levels yielded significantly lower conversion yields. Based on this optimization, a generic derivatization procedure was established: the reaction temperature was set at 70 °C with a reaction time of 1 h in acetonitrile in the presence of 1 mg/mL potassium carbonate and 1 mg/mL CAX-B. The reaction mixture was diluted 1:5 with water prior to LC-ESI-MS/MS analysis with an injection volume of 5 µL to ensure sharp and symmetric chromatographic peak shapes. Notably, no dilution or a 1:3 dilution resulted in asymmetric and broad peaks. A 1:5 dilution was the minimal dilution required to obtain proper peak shapes. The conversion under the selected conditions was >95% (disappearance of the intact OP acids as detected with an LC-MS/MS). The reaction between the OP acids and CAX-B along with the diagnostic ion of the core structure of each OP family derived from the ESI-MS/MS spectra are illustrated in Figure 1.

### 2.3. Structurally Informative High Resolution-MS/MS of Targeted OP Acids after Derivatization

#### 2.3.1. OP Acids Related to Nerve Agents (AMPAs)

**Figure 2:**
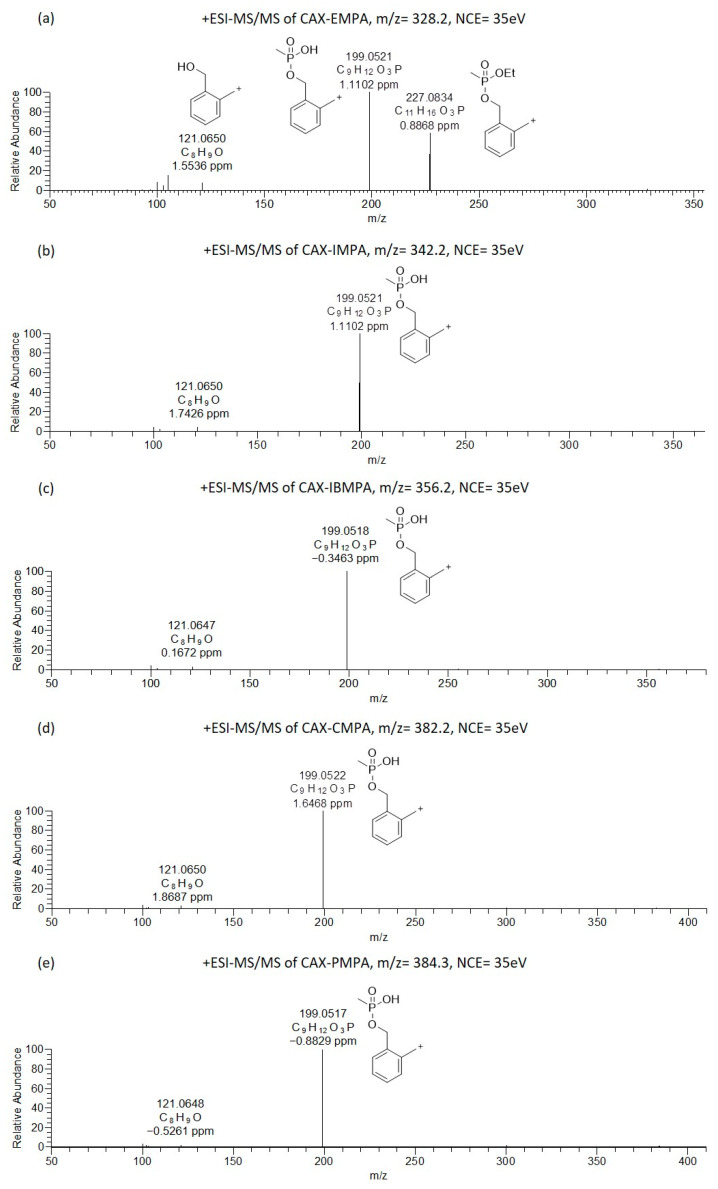
Oribitrap-ESI-MS/MS spectra of 10 ng/mL solutions of EMPA (**a**), IMPA (**b**), IBMPA (**c**), CMPA (**d**) and PMPA (**e**) after derivatization with CAX-B at a normalized collision energy (NCE) of 35 V (<3 ppm error).

**Figure 3:**
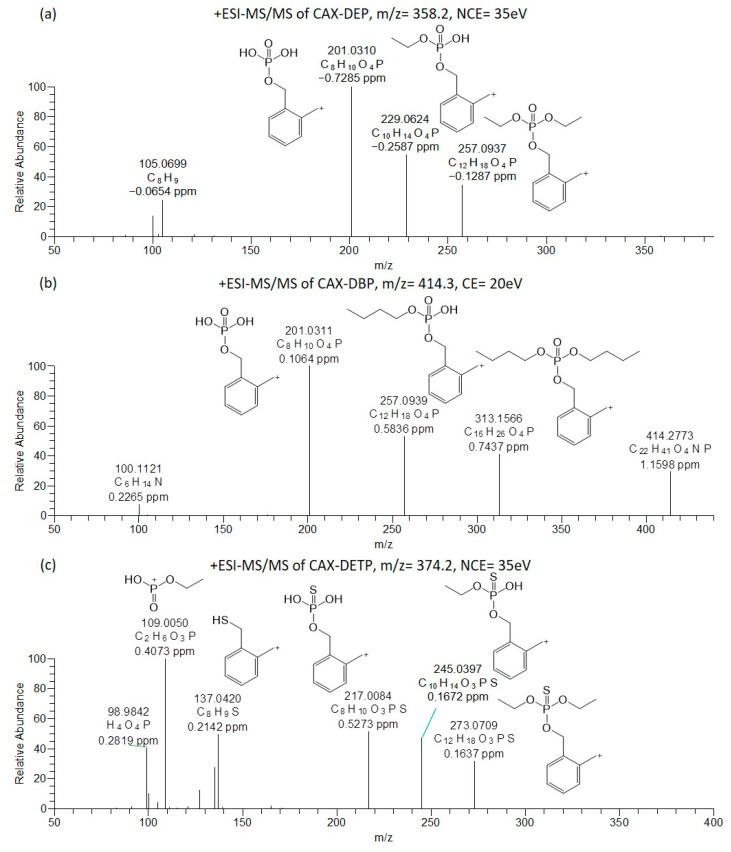
Oribitrap-ESI-MS/MS spectra of 10 ng/mL solutions of DEP (**a**), DBP (**b**), and DETP (**c**) after derivatization with CAX-B at a normalized collision energy (NCE) of 35 V, except DBP at a CE of 20 V (<3 ppm error).

Orbitrap-ESI-MS/MS experiments were executed for the OP-acids after derivatization by varying the collision energy (CE = 20–40 eV, NCE = 35 eV). Their ESI-MS/MS spectra were interpreted and enabled us to study the fragmentation pathways with the aim of seeking characteristic product ions indicative of the suborganophosphorus groups. The suggested product ion structures, depicted in Figure 2 and Figure 3, are based on the fragmentation rules we previously established [26]. Further steps were to develop and establish MRM transitions utilizing a Xevo-TQS instrument and to compare the identification sensitivity of the OP-acid prior to and after derivatization with CAX-B.

The Orbitrap-ESI-MS/MS spectra of the alkyl methylphosphonic acids after derivatization with CAX-B are illustrated in Figure 2. The ESI-MS/MS spectra of CAX-EMPA (*m*/*z* 328.2036) exhibited four product ions at *m*/*z* 227.0834, 199.0521, 121.0650, and 105.0700 (Figure 2a). One of the most intense product ions in the ESI-MS/MS spectra was observed at *m*/*z* 227.0834, an analyte—characteristic of the first product ion derived from the loss of the neutral triethylamine from the precursor ion. Another intense product ion was observed at *m*/*z* 199.0521, which was attributed to the loss of the triethylamine and ethylene groups. The ion at *m*/*z* 199.0521 was also observed in other OP acids related to chemical warfare agents (IMPA, IBMPA, CMPA, and PMPA). This ion is attributed to the loss of both the triethylamine and isopropylene, isobutylene, cyclohexylene, and 2,2-dimethyl-1-butylene in IMPA, IBMPA, CMPA and PMPA, respectively, and is indicative of the methylphosphonate core structure and provides information on the substructure of “unknown” OP acids.

A xylyl-based cation (similar to the product ion at *m*/*z* 227 in the ESI-MS/MS spectra of CAX-EMPA) was not observed in the ESI-MS/MS spectra of the CAX-B derivatives of IMPA, IBMPA, CMPA, and PMPA, due to the facile formation of two parallel dissociation processes resulting in trimethylamine and alkene loss. This is probably because alkyl substituents larger than ethyl tend to dissociate faster than ethyl substituents. This phenomenon was in accordance with our previous study on V-type nerve agents, where an O-Et cleavage was not observed in VX, but an O-^i^Bu cleavage was observed in Russian VX [27]. Another ion at *m*/*z* 121.0650 was attributed to the hydroxyl-methyl tropylium ion.

#### 2.3.2. OP Acids Related to Pesticides

Orbitrap-ESI-MS/MS experiments were carried out for the two dialkyl phosphates (DEP and DBP) and for the diethylthiophosphate (DETP) after derivatization with CAX-B. The MS/MS of the protonated molecules [M + H]^+^ was performed by varying the collision energy (20–40 eV) and at a normalized collision energy of 35 eV. The ESI-MS/MS spectra of the CAX-DEP, CAX-DBP, and CAX-DETP are shown in Figure 3. 

These three acids contain two ethyl chains (DEP, DETP) or two butyl chains (DBP) attached to oxygen. As assumed, the ESI(+)-MS/MS spectra of the derivatives (CAX-DEP *m*/*z* 358.2141, CAX-DBP, *m*/*z* 414.2768) revealed an ion attributed to the loss of a triethylamine from the [M + H]^+^ precursor ions to generate ions at *m*/*z* 257.0937 and 313.1566, respectively. In addition, ions were derived from the neutral losses of one and two ethylene groups in CAX-DEP (*m*/*z* 229.0624 and *m*/*z* 201.0310) and one and two butylene groups in CAX-DBP (*m*/*z* 257.0939 and 201.0311). The ion at *m*/*z* 201.0311 is indicative of the dialkyl phosphate core structure and provides information on the substructure of “unknown” OP acids.

The ESI(+)-MS/MS spectrum of DETP after derivatization with CAX-B revealed a high abundance ion attributed to the loss of triethylamine from the [M + H]^+^ precursor ion to generate an ion at *m*/*z* 273.0709. In addition, ions at *m*/*z* 245.0397 (loss of ethylene) and *m*/*z* 217.0084 (loss of two ethylene groups) were also observed. The ion at *m*/*z* 217.0084 is characteristic of the dialkyl thiophosphate core structure and is vital for determining the structure of “unknown” OP acids of the dialkyl thiophosphate substructure. Additionally, a low abundance, diagnostic ion at *m*/*z* 109.0050 [C_2_H_6_O_3_P]^+^ was observed. This ion is attributed to the monoethyl phosphate cation. The dissociation of two consecutive alkyl chains was in accordance with the experimental findings concerning fragmentations of phosphates and reported in the literature [20,28].

### 2.4. Sensitivity Enhancement after Derivatization with CAX-B Using a QqQ Instrument

**Figure 4:**
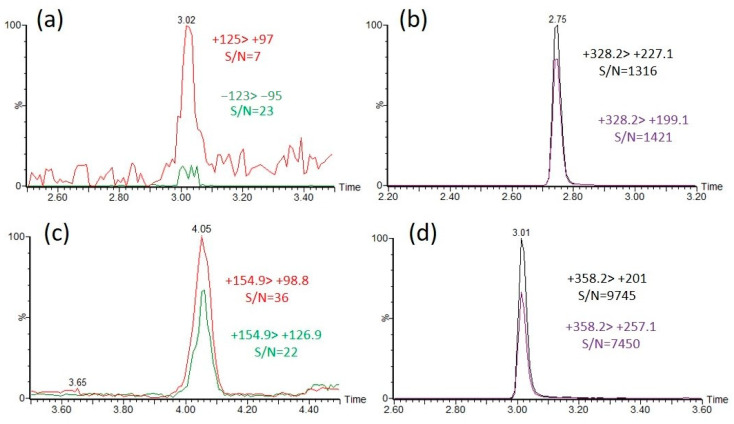
Sensitivity enhancement of the identification of EMPA and DEP by derivatization with CAX-B in pure acetonitrile. Overlaid extracted ion chromatograms (EICs) of the two dominant MRM transitions of EMPA, RT = 3.0 min (**a**) and DEP, RT = 4.0 min (**c**), and the derivatization products CAX-EMPA, RT = 2.7 (**b**) and CAX-DEP, RT = 3.0 min (**d**). A 10 ng/mL solution of EMPA and DEP was analyzed under neutral eluents with an LC-MS/MS. Then, 1 mg of CAX-B and 1 mg of K_2_CO_3_ were added, followed by the derivatization procedure and analysis with an LC-MS/MS under acidic eluents.

To compare the identification sensitivity between the intact OP acids and the CAX-B-derivatized OP acids, OP acids at concentrations of 10 ng/mL were derivatized with CAX-B to the corresponding OP derivatives, and the reaction mixtures were diluted 1:5 with water and analyzed with an LC-ESI-MS/MS in MRM mode. For the nonderivatized OP acids, the most intense MRM transition was observed with a signal-to-noise ratio (S/N) of 20–60. However, for CAX-OP-acid derivatives, two to five high-intensity MRM transitions were observed, with a signal-to-noise ratio (S/N) of 1000–3000. The MRM transition signal with the highest S/N ratio of the intact EMPA ((−)123 > 95) was 60 times lower than that of the MRM transition signal with the highest S/N ratio of the CAX-EMPA ((+)328 > 199) using an LC-ESI-MS/MS (Figure 4a,b), and the highest intensity MRM signal of the intact DEP ((+)155 > 99) was more than two orders of magnitude lower than that of the MRM signal intensity of CAX-DEP ((+)358 > 201) (Figure 4c,d). In the same manner, the highest intensity MRM transition signals of all the intact OP acids were compared, and the derivatization products of the OP acids were found to be substantially more sensitive by one to over two orders of magnitude.

In conclusion, CAX-B was found to be a powerful reagent for the derivatization of OP acids. Several OP-acid derivatives presented the production of an analyte—characteristic of the first product ion (as a xylyl-based cation) from the loss of neutral triethylamine from the precursor ion (EMPA, DEP, DBP, or DETP).

O-alkyl cleavages were observed in all intact OP acids in positive ion mode, generating several sequential product ions from the precursor ion [M + H]^+^. After derivatization of the OP acids with CAX-B, these O-alkyl cleavages were also observed; however, at a higher intensity due to the attached xylene-tag moiety, both in the precursor and product ions. In addition, the formation of the OP core characteristic ion is beneficial for the determination of the specific substructure (phosphonate/phosphate/thiophosphate) and is useful for the identification of “unknown” OP acids.

### 2.5. Method Evaluation

Method evaluation was performed in ACN. As this method is qualitative only, method performance was determined by evaluating the calibration curve linearity, limits of identification (LOIs), relative standard deviations (RSDs), and stability of CAX-OP-acid derivatives.

**Figure 5:**
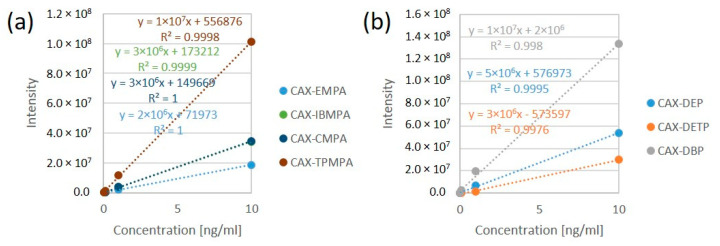
Linear calibration curves of CAX-OP-acid derivatives related to nerve agents (**a**) and pesticides (**b**). The derivatization reaction was carried out in ACN in the concentration range of 0.01–10 ng/mL.

The derivatization reaction for all OP acids was carried out in ACN in the concentration range of 0.01–10 ng/mL. All the calibration curves for the CAX-OP-acid derivatives were linear over the range of 0.01–10 ng/mL (except CAX-EMPA, which was linear over the range of 0.1–10 ng/mL), with a coefficient of determination (R^2^) greater than 0.99, as shown in Figure 5.

At a spiking concentration of 0.01 ng/mL in ACN, all CAX-OP acid derivatives showed at least two MRM signals with S/N > 3; hence, the identification limit was determined at the low ppt level. The stability of the CAX-OP-acid derivatives was also established with no observable dissociation after 24 h on the LC-MS tray (~25 °C). The repeatability of the method was determined by conducting three independent derivatizations at each concentration in the calibration curve and measuring the resulting relative standard deviations (RSDs) in the intensity of the most intense MRM signal for each analyte. For all seven CAX-OP acids, the RSDs were lower than 20%.

### 2.6. Application in Real-World Matrices

After demonstrating the ability of the method to convert OP acids into their corresponding CAX-OP esters, we tested its performance on real-world matrices. The reactivity of CAX-B was assessed toward OP acids in acetonitrile extracts of plant, concrete, and hair, which represent environmental and clinical matrices that OP nerve agents and pesticides may degrade in, resulting in low amounts of OP-acids.

**Figure 6:**
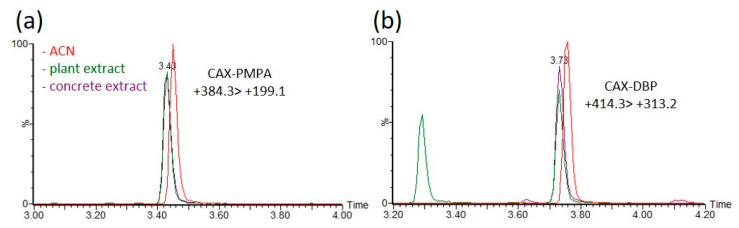
Matrix effects on the derivatization rate and ionization efficiency of the derivatization product of PMPA and DBP in acetonitrile extracts of different environmental matrices. Compared signal intensities of the dominant MRM transition (+384.3 > 199.1 for CAX-PMPA and +414.3 > 313.2 for CAX-DBP) of the derivatization product CAX-PMPA (**a**) and CAX-DBP (**b**) between spiked acetonitrile extracts of plants (green), concrete (purple) and spiked ACN (red). ACN and acetonitrile extracts of blank matrices were spiked with PMPA or DBP at a concentration of 1 ng/mL, followed by the instantaneous addition of 1 mg of CAX-B and 1 mg of K_2_CO_3_, stirring for 1 h at 70 °C prior to LC-ESI-MS/MS analysis. Response ratios lower or higher than 100% indicate signal suppression or enhancement.

Environmental matrices are well known to affect the ionization efficiency (enhancement or suppression) and to affect the derivatization rates in LC–MS analysis. Matrix effects were evaluated by comparing the acetonitrile extracts of a blank matrix spiked with OP-acids and pure acetonitrile solvent, followed by derivatization with CAX-B prior to analysis. Acetonitrile extracts of plants, hair and concrete were spiked with five OP acids (EMPA, PMPA, DEP, DBP, and DETP) at concentrations between 0.02 and 10 ng/mL, and the derivatization procedure was applied. The linearity and matrix effects were evaluated. The calibration curves for the derivatization product were linear in the range of 0.02–10 ng/mL, with a coefficient of determination (R^2^) greater than 0.99 (Figure S1). No interferences were observed at near retention times (Figure S2), and a signal suppression of ~20% and a slight decrease in RT (~0.5%) due to matrix effects were observed, as shown in Figure 6, which illustrates MRM signals of CAX-PMPA ((+)384 > 199) and CAX-DBP ((+)414 > 313) in ACN and in ACN extracts of plants and concrete. The LOIs of the OP-acids (EMPA, PMPA, DEP, DBP, and DETP) were 1–10 ng/mL and 0.02–0.2 ng/mL before and after derivatization, respectively (Table S1).

**Figure 7:**
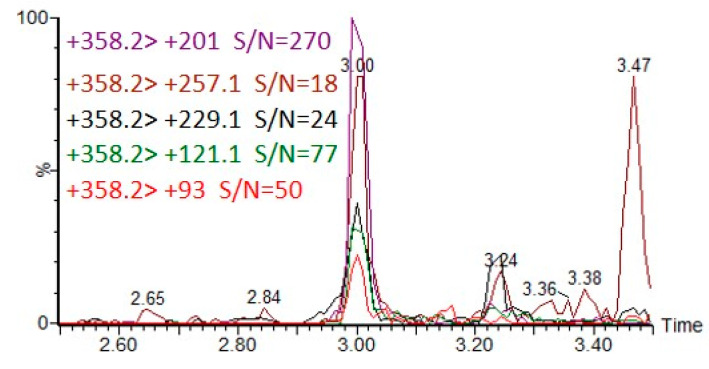
Five MRM transitions of CAX-DEP in LC-MS/MS analysis of 20 ng/mL DEP spiked into acetonitrile hair extract, followed by the derivatization procedure.

Figure 7 illustrates the extracted ion chromatograms (EICs) of five MRM transitions of CAX-DEP when DEP was spiked to a final concentration of 20 pg/mL in an acetonitrile extract of hair followed by derivatization with CAX-B and LC-ESI-MS/MS analysis.

## 3. Materials and Methods

### 3.1. Chemicals and Reagents

Six nerve agent-related hydrolysis products, EMPA, IMPA, IBMPA, CMPA, and PMPA, were synthesized in-house. All other chemicals were purchased from commercial suppliers. The three pesticide-related hydrolysis products, DEP, DBP, and DETP, the derivatizing agent CAX-B, and potassium carbonate were obtained from Sigma-Aldrich (St. Louis, MO, USA). Formic acid (MS grade), acetonitrile (MS grade), and water (MS grade) were obtained from Biolab (Jerusalem, Israel).

Pooled human head hair was collected from a barber shop, plant leaves were collected from lemon and orange trees, and a concrete block was collected from a construction site.

### 3.2. Sample Preparation

A stock solution (100 ng/mL) of EMPA, IMPA, IBMPA, CMPA, PMPA, DEP, DBP, and DETP was prepared in acetonitrile (ACN) and stored at −20 °C. This solution was further diluted in acetonitrile/acetonitrile extracts prior to derivatization to concentrations of 0.01–10 ng/mL.

#### 3.2.1. Derivatization Procedure

First, 0.5 mg of K_2_CO_3_ and 0.5 mg of the derivatizing agent CAX-B were added into an Eppendorf tube containing 500 μL of an acetonitrile/acetonitrile extract containing 5 pg to 5 ng of analytes. Then, the solution was stirred using an Eppendorf vortex mixer at 70 °C and 800 rpm for 1 h. The content was then diluted 1:5 with water and transferred into an LC conic vial prior to LC-ESI-MS/MS analysis.

#### 3.2.2. Spiking of Environmental Matrices Extracts

Plant leaves (5.2 g), human hair (2.2 g), and concrete (20.2 g) were vortexed with 28, 17, and 20 mL of acetonitrile, respectively, in a 50 mL polypropylene tube for 30 s and rested in a fume hood at room temperature for 85 min. Then, the acetonitrile extracts were filtered using a 0.22 µm PVDF filter into a fresh glass bottle and kept at 4 °C.

Each acetonitrile extract (500 µL) was spiked with the analytes and immediately subjected to the abovementioned derivatization procedure.

### 3.3. Instrumentation

#### 3.3.1. HPLC Method

Chromatographic separation was achieved using a reversed-phase column (Gemini C18, 3.0 μm, 150 mm, 2.1 mm ID by Phenomenex, Switzerland) at 40 °C with a flow rate of 0.3 mL/min. All injections were 5 μL.

For the separation of intact OP acids, the mobile phase consisted of 1 mM ammonium formate in water (A) and in methanol (B). For the separation of CAX-OP derivatization products, the mobile phase consisted of 0.1% formic acid in water (A) and methanol (B).

The gradient profile began with 100% mobile phase A and ramped linearly to 5% A over 6 min. Then, the flow was held at 5% A for 3.5 min, switched back to 100% A, and held for 5.5 additional minutes up to a total cycle time of 15 min to equilibrate the column for the next sample.

#### 3.3.2. LC-QqQ/MS/MS (MRM) Analysis

The LC-MS/MS system consisted of an Acquity UPLC I class system coupled to a Xevo TQ-S triple quadrupole tandem mass spectrometer (Waters Corporation, Milford, MA, USA), which was operated with a positive/negative ESI source in multiple reaction monitoring (MRM) mode. The MS/MS parameters were set as follows: capillary voltage, 0.6 kV; source temperature, 150 °C; desolvation temperature, 500 °C; desolvation gas flow, 800 L/h; cone gas flow, 150 L/h; and collision gas flow, 0.15 mL/min. The Intellistart feature in Waters MassLynx software (version 4.2 SCN986, Waters Corp., Milford, MA, USA) was used for determining the most intense MRM transitions and optimizing the cone voltages and collision energies.

##### LC-ESI-MS/MS Analysis of the Intact OP Acids

The OP acids related to the nerve agents EMPA, IBMPA, CMPA, and PMPA were analyzed in their intact forms to determine their responses with an RPLC-ESI(−/+)-QqQ-MS/MS before derivatization. Except for EMPA that yielded a protonated molecule [M + H]^+^ with a relatively abundant product ion at *m*/*z* 97, the OP acids yielded deprotonated molecules [M − H]^−^ with the two most abundant MRM transitions listed in Table 1.

The OP acids related to the pesticides DEP, DBP, and DETP were also analyzed in their intact forms to determine their responses with an RPLC-ESI(+)-QqQ-MS/MS before derivatization. These OP acids yielded protonated molecules [M + H]^+^ with the two most abundant MRM transitions listed in Table 1.

##### LC-ESI-MS/MS Analysis of CAX-B Derivatives

The detection and identification of all OP-acids after derivatization with CAX-B was performed in positive ion MRM mode using the most abundant MRM transitions listed in Table 2.

#### 3.3.3. LC-MS (Orbitrap Mass Spectrometry)

The LC-MS system consisted of an Agilent 1290 HPLC system (Palo Alto, CA, USA) connected to a Q-Exactive+ Orbitrap mass spectrometer (Thermo Fisher Scientific, Bremen, Germany) equipped with a heated electrospray ionization (HESI) ion source operated in positive ion mode.

The ESI ion source operating parameters were as follows: electrospray voltage was 1.25 kV; sheath gas flow rate was 45 (arbitrary units); auxiliary gas was 10 (arbitrary units); sweep gas was 2 (arbitrary units); auxiliary gas heater temperature was 400 °C; and the capillary temperature was 275 °C. MS and MS/MS parameters are listed in Table 3.

### 3.4. Comparison of the Sensitivity of the Intact OP Acids and CAX-OP Acids after Derivatization

OP acid solutions in ACN at a concentration range of 0.01–10 ng/mL were analyzed prior to derivatization in the negative and positive ion modes and after derivatization in the positive ion mode. The S/N ratios of the two most intense MRM transitions before and after derivatization were compared.

## 4. Conclusions

CAX-B was found to be an efficient derivatizing agent for increasing the sensitivity of all investigated OP acids in tandem mass spectrometry from complex matrices, such as concrete, plant, and hair extracts. The conversion of the OP acids to their corresponding derivatives improved the detection sensitivity by one to over two orders of magnitude compared with the detection of non-derivatized analytes in LC-ESI-MS/MS analysis and allowed for detection in the low ppt range. Moreover, its ease of use and the formation of product ions characteristic of the sub-OP group make it a good choice for the enhanced detection of targeted and “unknown” OP acids.

## Figures and Tables

**Table 1:** MRM parameters and chromatographic attributes of intact OP-acids.

OP-Acid	Formula	Precursor Ion (*m*/*z*)	Product Ion	Cone Voltage (V)	Collision Energy (eV)	Intensity Ratio	Retention Time (min)
EMPA	C_3_H_9_O_3_P	(−)123.0 (+)125.0	95.0 97.0	12	12 10	1 7.5	3.0
IBMPA	C_5_H_13_O_3_P	(−)151.1	95.0 77.0	52	14 20	5 1	5.4
CMPA	C_7_H_15_O_3_P	(−)177.1	95.0 79.0	12	20 32	25 1	6.3
PMPA	C_7_H_17_O_3_P	(−)179.1	95.0 79.0	12	16 32	23 1	6.9
DEP	C_4_H_11_O_4_P	(+)155.0	127.0 99.0	36	8 14	1 1.5	4.0
DBP	C_8_H_19_O_4_P	(+)211.1	155.0 99.0	22	6 10	1 3.5	7.5
DETP	C_4_H_11_O_3_PS	(+)171.0	115.0 143.0	26	12 8	1 1	4.8

**Table 2:** MRM parameters and chromatographic attributes of CAX-OP-acids.

CAX-OP-Acid	Formula	Precursor Ion (*m*/*z*)	Product Ion	Cone Voltage (V)	Collision Energy (eV)	Intensity Ratio	Retention Time (min)
CAX-EMPA	C_17_H_30_NO_3_P	(+)328.2	227.1 199.1 121.1 103.1	12	15 20 25 35	3 2.5 1 1.5	2.7
CAX-IBMPA	C_19_H_34_NO_3_P	(+)356.2	199.1 121.1 103.1	52	20 32 38	6 1 1	3.2
CAX-CMPA	C_21_H_36_NO_3_P	(+)382.3	199.1 121.1 103.1	12	20 30 40	6.5 1 1	3.3
CAX-PMPA	C_21_H_38_NO_3_P	(+)384.3	199.1 121.1 103.1	56	22 36 46	6 1.5 1	3.4
CAX-DEP	C_18_H_33_NO_4_P	(+)358.2	257.1 229.1 201.0 121.0 93.0	14	16 22 26 40 50	3 2 4 2 1	3.0
CAX-DBP	C_22_H_41_NO_4_P	(+)414.3	313.2 257.1 201.0 121.0 93.0	60	14 18 28 42 56	1 1.5 1 3.5 1.5	3.7
CAX-DETP	C_18_H_33_NO_3_PS	(+)374.2	273.1 217.0 137.0 109.0 91.0	20	16 28 32 26 48	5.5 1 2.5 3.5 2	3.2

**Table 3:** Q-Exactive+ Orbitrap Setup of Full-Scan MS and DIA Parameters in this study.

Full-Scan (“Full MS”) Mode:	
Resolution	70,000
AGC (automatic gain control) target	1 × 10^6^
maximum IT	100 ms
Scan range	*m*/*z* 50–500
DIA (data-independent acquisition) MS^2^ mode:	
Resolution	35,000
AGC target	5 × 10^5^
maximum IT	Auto
loop count	3
isolation window	*m*/*z* 1.0
NCE (normalized collision energy)/CE	35 V/20–40 V

## Data Availability

The data presented in this study supporting the results are available in the main text and Supplementary Materials. Additional data are available upon request from the corresponding authors.

## Supplementary Materials

The following supporting information can be downloaded at: https://www.mdpi.com/article/10.3390/molecules28166090/s1, Figure S1: Linear calibration curves of CAX-EMPA, CAX-PMPA, CAX-DETP and CAX-DBP in acetonitrile extracts of concrete (a) and plant (b); Figure S2: Chromatograms of 200 pg/mL CAX-EMPA (a), CAX-PMPA (b) and CAX-DETP (c) in concrete extract (green) over blank concrete extract (red); Table S1: Limits of identification (LOIs) of the OP-acids before and after derivatization in real-world matrices.

## Sample Availability

Not available.
