# Supplementary material for: Enhanced LC-ESI-MS/MS Sensitivity by Cationic Derivatization of Organophosphorus Acids
Source: Molecules. 2023 Aug 16;28(16):6090. doi: 10.3390/molecules28166090 (PMC10459995; doi:10.3390/molecules28166090)
Supplement: Supplementary file 1 [file molecules-28-06090-s001.zip › molecules-2536217-supplementary.pdf]

__Supplementary Material-__

"Enhanced LC–ESI–MS/MS Sensitivity by Cationic Derivatization of Organophosphorus Acids"

Figure S1. Linear calibration curves of CAX-EMPA, CAX-PMPA, CAX-DETP and CAX-DBP in acetonitrile extracts of concrete (a) and plant (b).

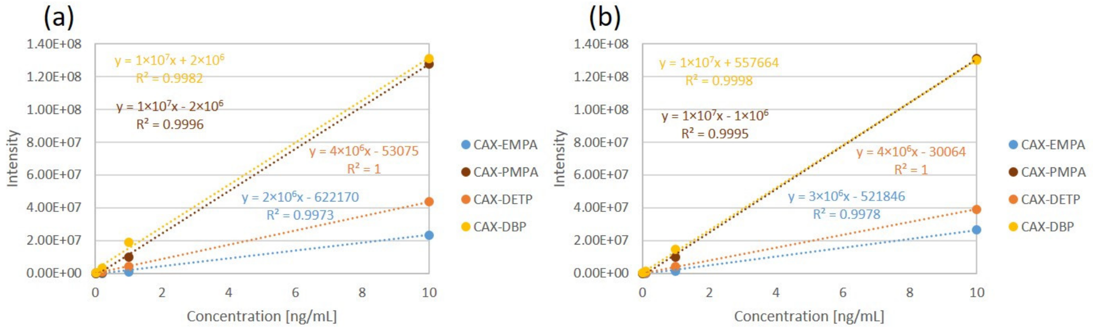

Figure S2. Chromatograms of 200 pg/mL CAX-EMPA (a), CAX-PMPA (b) and CAX-DETP (c) in concrete extract (green) over blank concrete extract (red).

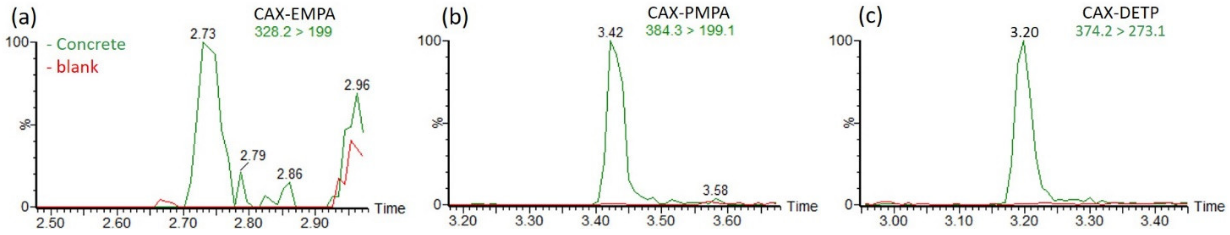

Table S1. Limits of identification (LOIs) of OP-acids before and after derivatization in real-world matrices.

Compound	LOI in Plant ext. (ng/mL)		LOI in Concrete ext. (ng/mL)		LOI in Hair ext. (ng/mL)	
	Before der.	After der.	Before der.	After der.	Before der.	After der.
**EMPA**	10	0.02	10	0.2	10	0.2
**PMPA**	10	0.1	1	0.2	10	0.2
**DEP**	10	0.02	10	0.02	10	0.02
**DBP**	10	0.02	10	0.02	10	0.02
**DETP**	10	0.1	10	0.02	10	0.02
